# Education and Health: Redrawing the Preston Curve

**DOI:** 10.1111/padr.12141

**Published:** 2018-04-14

**Authors:** Wolfgang Lutz, Endale Kebede

Progress in human health and life expectancy is closely associated with socioeconomic development. Better nutrition and greater affordability of health care associated with higher income have been widely considered as primary determinants of historical and contemporary mortality declines. McKeown's (1976) influential book on the modern rise of population attributed the secular mortality decline largely to improving standards of living. Reviewing mortality improvements in Britain during the second half of the nineteenth century and the beginning of the twentieth, he argued that medical discoveries were of little consequence for the significant gains in survival during this period. His analysis served as a reference point of Preston's (1975) article, which is the focus of the present study. Preston showed that the global pattern over the twentieth century indicates an upward shift of the curve that links GDP per person on the horizontal axis and life expectancy on the vertical (Figure 1).^1^ Preston interpreted this shift as the effect of medical progress and health care over and above the effect of income. In many of the studies of this issue that followed Preston's lead, the assumption that income is the most important driver of mortality decline has been an unquestioned starting point.

**Figure 1:**
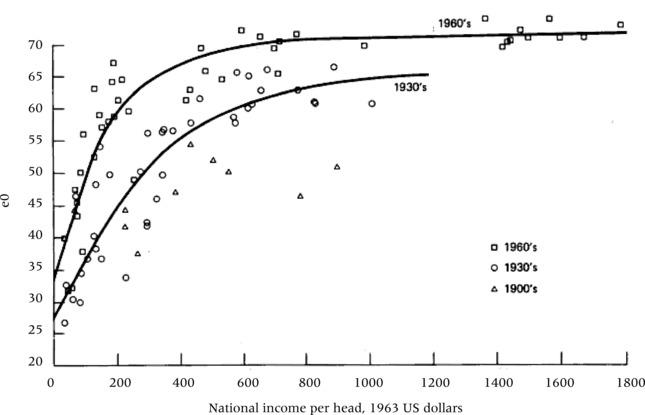
Scatter‐diagram of relations between life expectancy at birth (e0) and national income per head for nations in the 1900s, 1930s, and 1960s (from Preston 1975)^1^

A very different picture was drawn by Caldwell in a 1986 article on routes to low mortality in poor countries. Based on a major Rockefeller Foundation study on Kerala (India), Sri Lanka, and Costa Rica, Caldwell discussed the factors that led to breakthrough mortality declines in those populations as opposed to others and identified “female autonomy,” which he saw largely as a function of female education, as the single most important factor, together with efficient local health services. He also stated that his conclusion that low mortality does not come as an unplanned spinoff from economic growth was “out of step with today's dominant economic and political ideologies in the development field” (Caldwell 1986, p. 209). And this still seems to be the case three decades later, despite the fact that more recent research points to the overriding importance of education and the associated cognitive changes affecting risk perception, planning horizon, and access to information promoting health‐related behaviors and use of health care facilities (Baker et al. 2011; Lutz and Skirbekk 2014).

The question whether income or education is the more important determinant of global health and mortality decline is relevant for setting policy priorities in both developing and industrialized countries. The answer is of immediate concern in choosing between programs that directly promote economic growth and those that focus on enhancing school enrollment and quality of schooling. In an ideal world one would choose both in addition to good local health services but, in reality, even in rich countries there are budgetary constraints that require policymakers to set priorities. For helping to set these priorities, it is necessary to assess the relative importance of both factors. The question of relative importance is the focus of what follows. We address the issue at the macro level by plotting a modification of the Preston curve in which GDP per person is replaced by mean years of schooling among the adult population. This is done both for life expectancy at birth and for child mortality, and in both cases educational attainment explains the pattern better than GDP per person. This redrawing of the curve is complemented by a multivariate analysis to quantitatively assess the relative difference of the two effects.

## The Preston curve and its perception

In 1975 Samuel Preston published an influential paper, “The changing relation between mortality and level of economic development,” in which he plots the global relationship between GDP per capita and life expectancy at different points in time. He finds that over time the curve that depicts the relationship has moved upward, implying that a similar level of income is associated with higher life expectancy at later points in time (Preston 1975). He attributes this extra gain to medical progress. This view of the relationship, known as the Preston curve, has since become widely cited. Although Preston was cautious in interpreting this relationship as an association and not necessarily a causal one, subsequent interpretations of this relation often had no doubt that it was based on direct causation (e.g., Pritchett and Summers 1996).

In a more elaborate follow‐up study, Preston (1980) introduced literacy and calorie supply in addition to GDP per person into regression models to explain differences in levels of life expectancy for 36 countries in 1940 and 120 countries in 1970. The estimated coefficients showed similar patterns for both times, and literacy was highly significant. In Preston's words: “The coefficients indicate that a 10 percentage point increase in literacy is associated at both points with a gain in life expectancy of approximately 2 years, and that a 10 percent gain in national income by itself increases life expectancy by approximately one‐half year” (Preston 1980, p. 306). These interesting findings, however, were largely overlooked in subsequent research on development and mortality.

This apparent positive association between income and health has given rise to the widespread view among development economists that increased wealth leads causally to increased health. In the extreme, Pritchett and Summers (1996) argue that focusing on economic growth in developing countries will lead directly to reductions in infant mortality rates and gains in life expectancy. While many other economists hold a less definitive position and acknowledge drivers in addition to income, the possibility that the apparent empirical association between income and health could be largely spurious and not of a causal nature has not been considered in this body of economic literature.

In 2007 an issue of the *International Journal of Epidemiology* was devoted to a reprint of Preston's 1975 article and several comments by distinguished scholars in the field (IEA 2007). In the contribution most relevant for our analysis, Bloom and Canning (2007) revisit the Preston curve and state that his paper “remains a cornerstone of both global public health policy and academic discussion of public health. Preston's paper illuminates two central ‘stylized facts’. The first is a strong, positive relationship between national income levels and life expectancy in poorer countries, though the relationship is non‐linear as life expectancy levels in richer countries are less sensitive to variations in average income. The second is that the relationship is changing, with life expectancy increasing over time at all income levels. … Although the basic facts set out by Preston are generally accepted, there is still a great deal of dispute about the mechanisms that lie behind the relationships and the policy implications we can draw from them” (p. 498).

Bloom and Canning also point to another body of literature in public health in which, for example, Cutler, Deaton, and Lleras‐Muney (2006) conclude that scientific and technical advances should be seen as “the ultimate determinant of health.” A further argument against focusing on income growth as the primary method of reducing the burden of ill health lies in the apparently very weak temporal association between periods of economic growth and periods of improvement in population health, suggesting that if the relationship were causal, it has long and variable lags. While rising incomes imply greater resources for society, these resources need not necessarily be spent in ways that improve health. While Bloom and Canning do not question the basic assumption that income growth and health are closely linked, they add a cautionary sentence that is the starting point for our study: “Although there is a strong case for the direct effect of income on health due to nutrition and health interventions becoming more affordable, it may be that income is also acting as a proxy for a wider measure of socioeconomic status and development and that the causal effect is due to other mechanisms, for example, education” (p. 498).

In a more recent assessment of the Preston curve, Mackenbach and Looman (2013) find that for European countries increases in life expectancy after 1960 have been accompanied by a much smaller upward shift in the curve than previously. They attribute this to a changing pattern of causes of death away from infectious diseases and conclude that “declines in mortality from cardiovascular disease were mainly attributable to increases in national income.” This conclusion seems to be reached simply by eliminating medical progress as a dominating reason and assuming that the only other possible determinant of mortality was income, which thus should be the cause of this decline. The possibility of another driver that jointly determines income and health and causes their correlation was not considered.

In this article, we address the hypothesis that the apparent statistical association between income and health—as described by the Preston curve—could in fact be a largely spurious association resulting from the fact that improving educational attainment is a key determinant of both better health and rising incomes. Before doing so, we discuss the issue of causality.

## On causality

The question of the causal nature of the effects of education and income on health has attracted much attention and controversy. It is a question that needs to be addressed in order to rule out the possibility that the associations that are being interpreted could also be spurious. The question has important policy implications. If, for instance, the empirical association between income and health is not directly causal but rather due to a third factor such as education, then an increase in income—e.g., through policies directly aiming at economic growth—would not result in the expected health improvements unless educational attainment also improved simultaneously. The same is true *mutatis mutandis* for the association between educational attainment and health.

Causality in the social sciences has to be viewed differently than in the natural sciences because human behavior is culturally embedded and what is found to be direct causation of behavior in a given setting cannot be assumed to have universal predictive power for all societies and all times. Inspired by the comprehensive review of causality in demography by Ní Bhrolcháin and Dyson (2007), Lutz and Skirbekk (2014) introduced the notion of “functional causality” in the context of “intervention sciences” (Lutz and Striessnig 2015; Pearl 2000). Intervention sciences are the social and economic sciences that try to understand how the most important forces of change in a society function in order to predict the future evolution of the social system. Such conditional predictions about future trends can be based on the assumption of no intervention or of alternative interventions and their likely consequences.

To establish functional causality, three criteria have to be met: (i) there must be strong empirically observed associations between the two factors studied; (ii) there must be a plausible narrative about the mechanisms through which one force influences the other; and (iii) other obvious competing explanations of the observed association should be ruled out. Examples of such competing explanations are self‐selection, reverse causality, and joint determination by a third force (Lutz and KC 2011; Lutz and Skirbekk 2014). Lutz and Skirbekk (2014) give a comprehensive overview of dozens of relevant studies on the topic and conclude that it is justified to assume functional causality for the global‐level relationship between educational attainment and health/mortality over the twentieth and twenty‐first centuries.

Several global assessments of the relationship between health and mortality (Baker et al. 2011; KC and Lentzner 2010; Pamuk, Fuchs, and Lutz 2011) show that, on all continents and at different levels of socioeconomic development, the less‐educated segments of the population have significantly higher mortality and morbidity than those who are better educated. In virtually all countries, children of better‐educated mothers experience lower mortality. But since better‐educated people also tend to live in richer households, the question arises: what is more important for child survival in developing countries, mothers’ education or household income/wealth? This question has been the focus of studies using the largest available individual‐level data set by pooling the samples of Demographic and Health Surveys (DHS) in 43 developing countries (Fuchs, Pamuk, and Lutz 2010; Pamuk, Fuchs, and Lutz 2011). Using multi‐level regression models, analysis of the relative effects of mother's education and economic resources on infant mortality at the family, community, and country levels shows that the effect of education clearly dominates over income/wealth at all levels. The empirical evidence is equally compelling for the effect of education on adult mortality. In virtually all countries for which data exist, better‐educated people have higher life expectancies (Caselli et al. 2014). The differences vary in extent among countries and are generally greater for men than for women. Among industrialized countries, differences tend to be lowest in Southern European countries and are highest in Eastern Europe. In Russia, the difference between the highest and lowest education groups among adult men is up to 12 years (Caselli et al. 2014). It has also been shown that the overall decline in life expectancy that Russian men experienced over the 1990s was driven by a strong decline among the lower education groups whereas the highest groups continued to enjoy moderate increases (Shkolnikov et al. 2006). In virtually all industrialized countries for which data are available, the education differentials in adult mortality increased over time despite improving health care coverage in most countries (Caselli et al. 2014). One explanation for this pattern lies in the increasing importance of lifestyle‐related factors for which education seems to be more important than the health care system.

There is a plausible narrative of causation that is founded in brain research. It has been demonstrated that literacy and education in general enhance the synaptic density in relevant parts of our brains and thus makes us physiologically different for the rest of our lives (Kandel 2007). It has also been shown in a controlled experiment among illiterate Indian young adult men that the sub‐sample who was taught how to read and write had lasting structural changes in their brains after six months of learning that are associated with executive functioning and cognitive abilities (Baker, Salinas, and Eslinger 2012; Blair et al. 2005; Brinch and Galloway 2012; Skeide et al. 2017). Neurocognitive and neuroimaging studies have shown strong associations between adaptive changes in the brain and learning experiences in the classroom (Lewis et al. 2009; Welberg 2009). These changes have been shown to be associated with ability for abstract thinking, time preference, and the capacity to plan for the future (Cutler and Lleras‐Muney 2010; Kenkel 1991; Van der Pol 2011; Heckman, Humphries, and Veramendi 2017). These cognitive changes and resulting changes in behavior have relevance for health outcomes. In this context, it should also be stressed that the large number of studies that focus on natural experiments for the education/health link by looking at changes in compulsory schooling by a year do not seem to be a promising route because they do not refer to a plausible causal pathway. When young people are forced to stay in school against their will for ten rather than nine years, this is unlikely to result in a significant change in their overall brain functioning that would be expected to have direct health effects. But such relevant changes have been shown for major cognitive transitions such as from illiterate to literate or from compulsory to post‐secondary education (Cutler and Lleras‐Muney 2010; Peters et al. 2006; Skeide et al. 2017).

Another concern in the context of assessing functional causality is the possibility of reverse causality, which is also implicit in the notion of simultaneity. This can pose a difficult challenge if there is reason to assume simultaneous influences going in both directions. Models of Granger causality can be used to sort out the temporal sequence of possible effects that can then be the basis of causal inference based on the principle that the cause needs to precede the effect. But in the case of education such models are unnecessary because the temporal sequence can be identified *a priori*. Schooling tends to happen early in the life course, and it is only the human capital (education stock) at adult ages that is expected to have consequences for health‐related behavior. The time lag between when the schooling happens (education flow) and when the resulting stock of human capital influences health can be many decades and, when we study the education/health differentials for people above age 70, even half a century. Hence, once a proper distinction is made between education flow and educational attainment, there is no way in which period conditions at time *t* (including income, medical inventions, etc.) can influence the schooling that occurred decades before.

**Figure 2:**
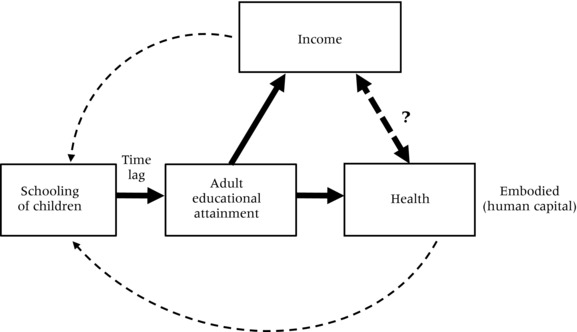
Triangular interactions between education flows and stocks, health, and income

With respect to the possibility that the empirical association is caused by a third factor, different pathways of causation need to be distinguished. Figure 2 shows the possible interactions between education, health, and income in the form of a triangle with education and health at the base and income at the top. The cognitive skills associated with adult educational attainment and a person's health status are closely interwoven and both embodied in individuals. A certain minimum level of physical health is necessary for a child to develop mentally and to be able to attend school. Particularly in developing countries, school absenteeism due to poor health of the children themselves or of family members for whom they must care or for whose lost economic output they must compensate is a serious handicap for improving levels of education and, consequently, the building of key cognitive skills and raising learning outcomes. Similarly, children often bring home from school knowledge, attitudes, and access to information relevant to the health and even survival of themselves and their family members.

Better health and longer lives are not only closely linked to education and cognitive capacity but also in turn directly affect economic growth. A growing literature shows that health is both a direct source of human welfare and a driver of income growth (Bloom and Canning 2008). In particular, three mechanisms have been defined: better health leading to higher labor productivity, better childhood health leading to better school attendance and cognitive development, and a longer expected life span leading to more savings and investment. Empirical studies also find that good health has a positive, sizable, and statistically significant effect on aggregate output, even controlling for the experience of the work force (Bloom, Canning, and Sevilla 2003). The WHO‐sponsored report of the Commission on Macroeconomics and Health (Sachs 2001) also highlighted the importance of basic health for poverty reduction by showing how the burden of diseases in some low‐income countries, especially in sub‐Saharan Africa, is a barrier to economic growth and therefore should be addressed in any comprehensive development strategy.

Finally, the effect of improving educational attainment and quality of education has long been a part of economic growth theory. Past empirical efforts to demonstrate this effect on the basis of aggregate time series have been hampered by the lack of appropriate data on educational attainment (de la Fuente and Doménech 2006). More recent assessments based on full educational attainment distributions by age cohorts demonstrate the consistently positive and significant effect of human capital on economic growth (Crespo Cuaresma, Lutz, and Sanderson 2014; Lutz, Crespo Cuaresma, and Sanderson 2008). The same has been shown with respect to quality of education for countries where such data exist (Hanushek and Woessmann 2008).

## Updating the global empirical analysis for 1970–2010

To explore the association between educational attainment, income, and mortality across time and space, we employ a balanced panel of 174 countries (both developed and developing) over the period 1970–2010 in five‐year intervals. Following the logic of Preston's 1975 and 1980 papers, we first present graphical presentations of the bi‐variate relationship between GDP per person and life expectancy, followed by multivariate statistical analyses. We also study the pattern with respect to child mortality. While the dichotomous variable of literacy was the only one available to Preston, we use mean years of schooling of the adult population aged 15 and older in the case of life expectancy and of women aged 20–39 in the case of child mortality.

### Data

Data were obtained mainly from two sources. Country‐level indicators of educational attainment for the years 1970–2015 were extracted from the Wittgenstein Centre Data Explorer (WIC 2015). Panel data on income and mortality were obtained from the World Development Indicators (World Bank 2017). Merging the two datasets gave us our panel of data with only a few missing data for some countries in earlier years. Following Preston's original design, income is measured as GDP per person (2010 constant USD). For the multivariate analysis, we also performed sensitivity runs where PPP (purchasing power parity) per person was used instead of constant 2010 USD.

### Descriptive analysis

**Figure 3:**
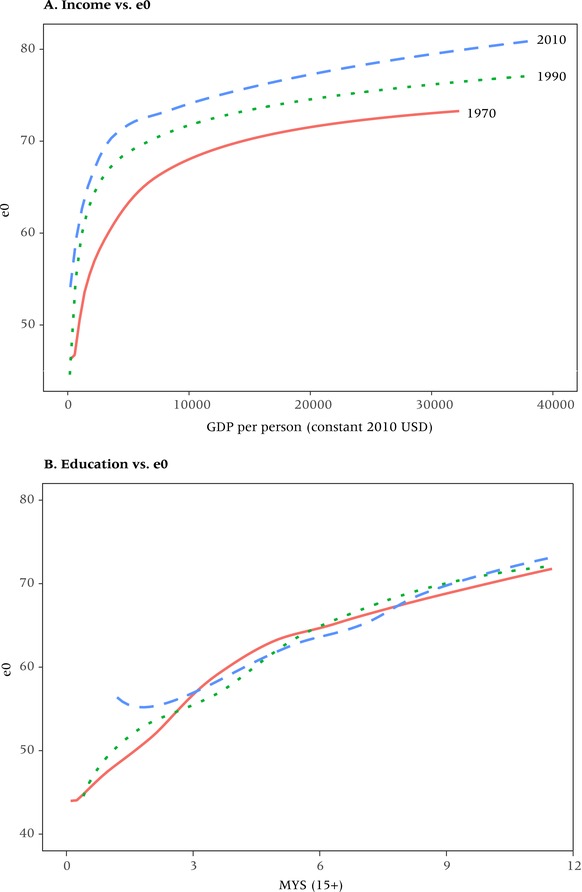
Relationship between real GDP per person (constant USD) and life expectancy at birth (Panel A) and between mean years of schooling (MYS) of the adult population aged 15+ and life expectancy at birth (Panel B), 1970, 1990, and 2010 NOTE: 174 countries, lines show fitted splines.

Figure 3 presents a visual analysis of the cross‐country associations between income, educational attainment, and life expectancy for the years 1970, 1990, and 2010, similar to the way in which Preston (1975) plotted life expectancy against GDP per person for the 1930s and 1960s. In Panel A, the plot of life expectancy at birth against GDP per person for 1970, 1990, and 2010 closely resembles the pattern of the original Preston curve (Figure 1). The curve clearly continues to move upward over time. This, according to Preston, is due to the factors other than income that he assumed to be mostly related to medical progress and health care. Panel B of the figure shows an isomorphic curve with the only difference that GDP per person is replaced by mean years of schooling of the adult population (MYS15+). The resulting pattern, however, differs considerably in that rising educational attainment seems to explain rising life expectancy much better than GDP per person. The association looks much more linear without a leveling off at higher levels, and there is very little upward shift that would indicate an unexplained gap that needs to be explained by medical progress.

**Figure 4:**
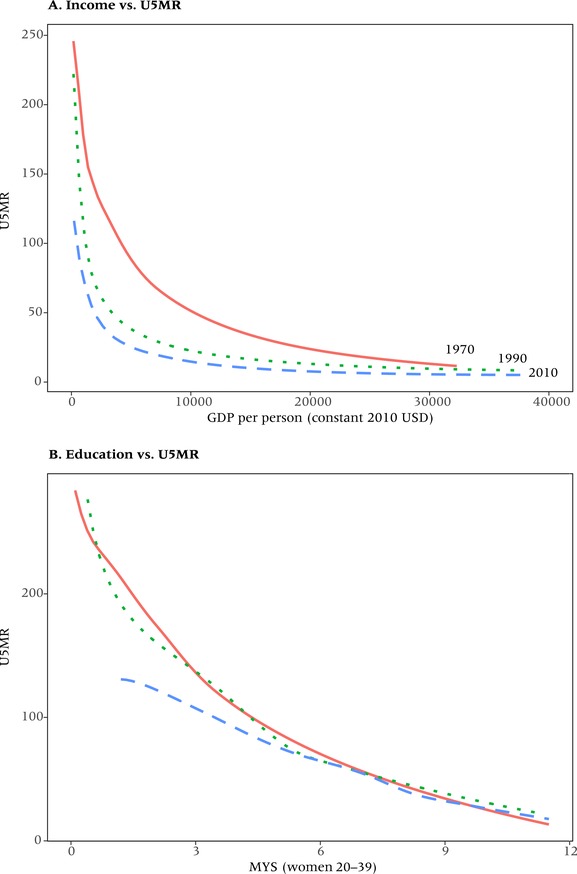
Relationship between real GDP per person (constant USD) and child mortality (U5MR) (Panel A) and between mean years of schooling (MYS) of women aged 20–39 and child mortality(Panel B), 1970, 1990, and 2010 NOTE: 174 countries, lines show fitted splines.

Figure 4 shows the corresponding pair of curves in which life expectancy is replaced by child mortality (aged 0–4) in order to see whether the previous pattern also holds for this mortality rate, which is often more easily influenced by targeted health interventions than is overall life expectancy. Here, mean years of schooling of women aged 20–39 is used as the education indicator. In Panel A, the plot of child mortality against GDP per person shows essentially the same pattern as for life expectancy vs GDP per person in Panel A of Figure 3. Panel B of the figure reveals that the relationship looks much more linear for education than for GDP per person; and for 1970–1990 there is no shift in the curves, with changes in mothers’ education evidently explaining declining child mortality much better than GDP per person. For 1990–2010, however, there is an interesting deviation from this general pattern, as child mortality in the high‐mortality/low‐education countries declines more rapidly than would be expected from the gains in mothers’ education over the same period. Evidently, this is the consequence of the massive international child health interventions in those countries over the past two decades. This shows that, at least with respect to child mortality, concerted public health efforts can lower mortality more than social development alone would predict.

### Multivariate analysis

Multivariate statistical analyses were conducted to explore the relative effects of education and income in explaining changing global mortality patterns. After conducting a Hausman test to choose between fixed‐effect and random‐effect specification for country‐level unobserved effects, the preferred fixed‐effect model was fitted and specified as follows:

(1) $$\;M_{it}=U_{i}\;+\beta_{1}Education_{it}+\beta_{2}GDP\;per\;person_{it}+\alpha \;\left(t\right)+\varepsilon it\;$$

(2) $$\alpha \;\left(t\right)=\alpha_{1}\;year_{1970}+\alpha_{2}year_{1975}+\alpha_{3}year_{1980}+\cdots +\alpha_{9}year_{2010}+\alpha_{10}$$

where $M_{it}$ is the mortality indicator (life expectancy at birth and child mortality) for country *i* at time *t*, $U_{i}\;$represent unobservable individual (country) heterogeneities,$\;\;Education_{it}$ is the education indicator (mean years of schooling for age 15+) for country iat time *t*, $\;GDP\;per\;person_{it}$ is GDP per person (at constant 2010 USD) for country *i* at time *t*; and α(t) controls for the year fixed‐effect and is specified as a binary variable for each year of observation.

A number of different models were estimated and, in addition to the main models shown in Tables 1 and 2, sensitivity analysis was performed as described below. For the sake of comparison, all variables were standardized (Z‐scores were calculated) before we fit models. The coefficients are thus interpreted, within a given country, as the gain in health (in standard deviation) for a standard deviation change in the independent variable. First, we estimated a country fixed‐effect panel model by including each explanatory variable separately: the unadjusted effect of each predictor is estimated and shown in column (1). Second, we estimated a model with both education and income indicators as shown in column (2). Finally, column (3) gives the full model with income, education, and both country and period fixed‐effects.

**Table 1:** Panel data regressions for the period 1970–2015 (population weighted) with country and time fixed‐effects as indicated (standardized coefficients), life expectancy at birth (standardized) as the dependent variable

	(1)	(2)	(3)
Variable	Unadjusted	Mutually Adj.	Main model
GDP per person (log)	.914***	.307**	.106
	(.234)	(.134)	(.091)
Education	.994***	.855***	.395***
	(.107)	(.104)	(.115)
Observations		1,276	1,276
R‐squared		.841	.884
Number of countries		149	149
Country fixed‐effects	Yes	Yes	Yes
Year fixed‐effects	No	No	Yes

^***^p<0.01, ^**^p<0.05, ^*^p<0.1.

NOTES: GDP per person is in constant 2010 USD and education is the mean years of schooling of the population aged 15+. Robust standard errors in parentheses.

**Table 2:** Panel data regressions for the period 1970–2015 (population weighted) with country and time fixed‐effects as indicated (standardized coefficients), under‐five child mortality (U5MR) (standardized) as the dependent variable

	(1)	(2)	(3)
Variable	Unadjusted	Mutually Adj.	Main model
GDP per person (log)	–.860***	–.004	.0772
	(.029)	(.118)	(0.127)
Education	–.923***	–1.214***	–1.016***
	(.034)	(.144)	(.130)
Observations		1,257	1,257
R‐squared		.824	0.831
Number of countries		147	147
Country fixed‐effects	Yes	Yes	Yes
Year fixed‐effects	No	No	Yes

^***^p<0.01, ^**^p<0.05, ^*^p<0.1.

NOTE: GDP per person is in constant 2010 USD and education is the mean years of schooling of women aged 20–39. Robust standard errors in parentheses.

The results for life expectancy given in Table 1 show high and significant unadjusted parameters for both income and education after controlling for country fixed‐effects, with the standardized education coefficient being somewhat higher. The picture changes substantially when income and education are entered in the same model, with the education coefficient becoming almost three times as large as the one for income. In the full model, which includes time fixed‐effects, income becomes insignificant while the education effect remains robust and highly significant.

The results with respect to infant mortality shown in Table 2 are quite similar to those for life expectancy. The difference is that income is already insignificant in the mutually adjusted model in column (2), and the coefficients for women's education (for ages 20–39) in that model and in the full model in column (3) are much higher than the comparable ones in Table 1 for overall mean years of schooling. Sensitivity runs using PPP income instead of constant USD (as in the original Preston paper) only marginally improved the coefficients for income, while the education effects remained robust and highly significant. In addition to the population‐weighted regressions shown here, we also ran the model giving every country equal weight. Since our dependent variables here—life expectancy and child mortality—reflect the averages of individual experiences and health‐related behaviors, the independent agents whose experience we are studying are individuals rather than countries, which makes population weighting more appropriate. In either case, the results of the unweighted regressions are qualitatively very similar, with the education coefficients slightly lower but still highly significant.

These multivariate results strongly confirm what the visual analysis above suggested: raising educational attainment is a much more important driver of increasing life expectancy and falling child mortality than income. As we discussed in the introduction, this finding should have significant implications for prioritizing policies aimed at improving health and longevity. Under a global perspective over the last half century, increasing educational attainment clearly has been the key factor in improving health, rather than increasing income as has frequently been claimed.

## Summary and conclusions

We revisited the influential 1975 paper by Preston on the relationship between income and life expectancy across most countries of the world for the 1930s and 1960s and extended the analysis to the period 1970–2015. We demonstrated that the distinct pattern identified by Preston, showing a strongly concave relationship and an upward shift of the curves, continued over the subsequent half century as assessed at the global level.

We then plotted the same kind of relationship replacing GDP per person with the mean years of schooling of the adult population to see whether educational attainment could be a better predictor of life expectancy than income. The associations turn out to be very different, with the curves becoming largely linear and overlapping. This suggests that educational attainment is a better predictor in the sense that its effect on life expectancy does not diminish at higher levels and, in particular, it does not leave an unexplained shift over time that has to be explained by other factors.

To validate this visual analysis, we conducted multivariate analyses on a balanced panel of 174 countries for 1970–2015, which in addition to GDP per person and mean years of schooling of the adult population included country and period fixed‐effects, and we performed sensitivity runs with alternative income indicators and weighting schemes. In all of the models the effect of educational attainment on life expectancy is highly significant in the expected direction, and the standardized coefficients are clearly larger than those of income.

To consider the possibility of a different pattern for the determinants of child mortality, we carried out the analysis separately for under‐5 mortality. Again, for the association with GDP per person there was strong non‐linearity and a shift of the curve over time that was particularly pronounced between 1970 and 1990. Viewed in relation to mean years of schooling of women aged 20–39, the relationship again was much more linear with virtually no shift between 1970 and 1990. Between 1990 and 2010 child mortality in the highest‐mortality countries declined more rapidly than suggested by the gains in mothers’ education. This is an indication that massive efforts by the international community and private donors in recent years to lower child mortality in some of the least developed countries were successful in doing so to a greater extent than would be expected from improving educational attainment alone. This was not equally the case with respect to adult mortality.

Where does this empirical evidence leave us with respect to testing the hypothesis that the empirical association between GDP and life expectancy depicted by the Preston curve and widely assumed in the literature is a spurious one, with education in fact driving both changes? The macro‐level evidence presented here strongly supports the view that this is a plausible hypothesis that deserves further elaboration. In our section on causality, we also assessed the different specified criteria for functional causality, including a strong empirical association, a valid narrative of the causal mechanism, and ruling out alternative explanations such as selectivity and reverse causality. This strong aggregate‐level finding should be further explored at the individual and community level under different cultural, social, and economic conditions.

Studies at the micro level are more easily conducted for child mortality than for adult mortality since the information can be provided in surveys by mothers. Multi‐level analyses of the determinants of child mortality across a large number of developing countries have shown that mothers’ education at every level of attainment was more important than wealth/income, was the dominating factor at the household and community level, and played a key role at the national level (Pamuk, Fuchs, and Lutz 2011). A comparable individual‐level study for adult mortality is much more difficult because there are no consistent data on adult deaths by education, income, and other relevant characteristics. Even in most industrialized countries with efficient vital registration systems, such micro‐level analysis will be difficult unless a comprehensive population register exists. With some extra effort, census data that have information on these characteristics can be linked with information about subsequent deaths. Where such matching studies have been conducted, they all show significant mortality differentials by level of education (Caselli et al. 2014). But such studies often lack the income information for comparative analysis. For developing countries, the data challenges are much greater and—possibly except for the cases of demographic surveillance systems—probably insurmountable at present.

The global time series analysis of national data strongly suggests that the apparent positive association between health and income can largely be attributed to increasing educational attainment, which at the same time leads to rising incomes (Lutz, Crespo Cuaresma, and Sanderson 2008) and better health outcomes. While additional individual‐level analysis of this issue is needed, the patterns presented here suggest that education should be considered a policy priority for improving global health.

## Notes

1 Figure originally published in Samuel H. Preston, “The changing relation between mortality and level of economic development,” *Population Studies*, Vol. 29, No. 2 (Jul., 1975), pp. 231–248, copyright © Population Investigation Committee, reprinted by permission of Taylor & Francis Ltd, http://www.tandfonline.com on behalf of Population Investigation Committee.
